# Does training improve diagnostic accuracy and inter-rater agreement in applying the Berlin radiographic definition of acute respiratory distress syndrome? A multicenter prospective study

**DOI:** 10.1186/s13054-017-1606-4

**Published:** 2017-01-20

**Authors:** Jin-Min Peng, Chuan-Yun Qian, Xiang-You Yu, Ming-Yan Zhao, Shu-Sheng Li, Xiao-Chun Ma, Yan Kang, Fa-Chun Zhou, Zhen-Yang He, Tie-He Qin, Yong-Jie Yin, Li Jiang, Zhen-Jie Hu, Ren-Hua Sun, Jian-Dong Lin, Tong Li, Da-Wei Wu, You-Zhong An, Yu-Hang Ai, Li-Hua Zhou, Xiang-Yuan Cao, Xi-Jing Zhang, Rong-Qing Sun, Er-Zhen Chen, Bin Du, for the China Critical Care Clinical Trial Group (CCCCTG)

**Affiliations:** 1 Medical ICU, Peking Union Medical College Hospital, 1 Shuai Fu Yuan, Beijing, 100730 People’s Republic of China; 2 Department of Emergency Medicine, The First Affiliated Hospital of Kunming Medical University, 295 Xichang Street, Kunming, 650032 People’s Republic of China; 3 Department of Critical Care Medicine, First Affiliated Hospital, Xinjiang Medical University, 1 Liyushan Road, Urumqi, 830054 People’s Republic of China; 4 Department of Critical Care Medicine, The First Affiliated Hospital, Harbin Medical University, 23 Youzheng Street, Harbin, 150001 People’s Republic of China; 5 Department of Critical Care Medicine, Tongji Hospital, Tongji Medical College, Huazhong University of Science & Technology, 1095 Jiefang Road, Wuhan, 430030 People’s Republic of China; 6 Department of Critical Care Medicine, The First Affiliated Hospital of China Medical University, 155 North Nanjing Street, Shenyang, 110001 People’s Republic of China; 7 Department of Critical Care Medicine, West China Hospital, Sichuan University, 37 Guoxue Alley, Chengdu, 610041 People’s Republic of China; 8 Department of Critical Care Medicine, The First Affiliated Hospital, Chongqing Medical University, 1 Youyi Road, Yuanjiagang, Chongqing, 400016 People’s Republic of China; 9 Department of Critical Care Medicine, Hainan Provincial People’s Hospital, No. 19 Xiuhua Road, Haikou, 570311 People’s Republic of China; 10 Department of Critical Care Medicine, Guangdong General Hospital, 106 Zhongshan Er Road, Guangzhou, 510080 People’s Republic of China; 11 Department of Emergency and Critical Care Medicine, The Second Hospital of Jilin University, 18 Ziqiang Street, Changchun, 130041 People’s Republic of China; 12 Department of Critical Care Medicine, Fuxing Hospital, Capital Medical University, A20 Fuxingmenwai Street, Beijing, 100038 People’s Republic of China; 13 Department of Critical Care Medicine, Hebei Medical University Fourth Hospital, 12 Jiankang Road, Shijiazhuang, 050011 People’s Republic of China; 14 Department of Critical Care Medicine, Zhejiang Provincial People’s Hospital, 158 Shangtang Road, Hangzhou, 310014 People’s Republic of China; 15 Department of Critical Care Medicine, The First Affiliated Hospital of Fujian Medical University, 20 Chazhong Road, Fuzhou, 350005 People’s Republic of China; 16 Department of Critical Care Medicine, Beijing Tongren Hospital, Capital Medical University, 2 Chongwenmennei Street, Beijing, 100730 People’s Republic of China; 17 Department of Critical Care Medicine, Qilu Hospital, Shandong University, 107 Wenhua Xi Road, Jinan, 250012 People’s Republic of China; 18 Department of Critical Care Medicine, Peking University People’s Hospital, 11 Xizhimen South Street, Beijing, 100044 People’s Republic of China; 19 Department of Critical Care Medicine, Xiangya Hospital, Central South University, 87 Xiangya Road, Changsha, 410008 People’s Republic of China; 20 Department of Critical Care Medicine, The Affiliated Hospital of Inner Mongolia Medical College, 1 Tongdao North Street, Huhhot, 010050 People’s Republic of China; 21 Department of Critical Care Medicine, Affiliated Hospital of Ningxia Medical University, 804 Shengli South Street, Yinchuan, 750004 People’s Republic of China; 22 Surgical ICU, Department of Anesthesia, Xijing Hospital, 127 Chang Le Xi Road, Xi’an, 710032 People’s Republic of China; 23 Surgical ICU, The First Affiliated Hospital of Zhengzhou University, 1 Jianshe Road, Zhengzhou, 450052 Henan People’s Republic of China; 24 Ruijin Hospital, Shanghai Jiao Tong University, No. 197 Ruijin Er Road, Shanghai, 200025 People’s Republic of China

**Keywords:** Acute respiratory distress syndrome, Chest radiograph, Diagnostic accuracy, Inter-rater variability

## Abstract

**Background:**

Poor inter-rater reliability in chest radiograph interpretation has been reported in the context of acute respiratory distress syndrome (ARDS), although not for the Berlin definition of ARDS. We sought to examine the effect of training material on the accuracy and consistency of intensivists’ chest radiograph interpretations for ARDS diagnosis.

**Methods:**

We conducted a rater agreement study in which 286 intensivists (residents 41.3%, junior attending physicians 35.3%, and senior attending physician 23.4%) independently reviewed the same 12 chest radiographs developed by the ARDS Definition Task Force (“the panel”) before and after training. Radiographic diagnoses by the panel were classified into the consistent (*n* = 4), equivocal (*n* = 4), and inconsistent (*n* = 4) categories and were used as a reference. The 1.5-hour training course attended by all 286 intensivists included introduction of the diagnostic rationale, and a subsequent in-depth discussion to reach consensus for all 12 radiographs.

**Results:**

Overall diagnostic accuracy, which was defined as the percentage of chest radiographs that were interpreted correctly, improved but remained poor after training (42.0 ± 14.8% before training vs. 55.3 ± 23.4% after training, *p* < 0.001). Diagnostic sensitivity and specificity improved after training for all diagnostic categories (*p* < 0.001), with the exception of specificity for the equivocal category (*p* = 0.883). Diagnostic accuracy was higher for the consistent category than for the inconsistent and equivocal categories (*p* < 0.001). Comparisons of pre-training and post-training results revealed that inter-rater agreement was poor and did not improve after training, as assessed by overall agreement (0.450 ± 0.406 vs. 0.461 ± 0.575, *p* = 0.792), Fleiss’s kappa (0.133 ± 0.575 vs. 0.178 ± 0.710, *p* = 0.405), and intraclass correlation coefficient (ICC; 0.219 vs. 0.276, *p* = 0.470).

**Conclusions:**

The radiographic diagnostic accuracy and inter-rater agreement were poor when the Berlin radiographic definition was used, and were not significantly improved by the training set of chest radiographs developed by the ARDS Definition Task Force.

**Trial registration:**

The study was registered at ClinicalTrials.gov (registration number NCT01704066) on 6 October 2012.

**Electronic supplementary material:**

The online version of this article (doi:10.1186/s13054-017-1606-4) contains supplementary material, which is available to authorized users.

## Background

The American-European Consensus Conference (AECC) definition of acute respiratory distress syndrome (ARDS) published in 1994 [1] has been widely adopted. However, limitations of this definition have been recognized, such as poor inter-observer reliability in identifying bilateral infiltrates consistent with pulmonary edema via chest X-rays [2–5]. The updated Berlin definition modified the previous radiographic criterion to require not only bilateral infiltrates but also the exclusion of effusion, lobar/lung collapse, or nodules [2]. It is unclear whether the modification has improved the reliability of chest X-ray interpretation. Recently, Bellan and colleagues reported in an international, multicenter, prospective cohort study that intensivists could only recognize 34.0% of ARDS at the time of fulfillment of ARDS criteria [6]. Although the exact reasons for the high number of under-recognized cases of ARDS might be multifactorial, the inappropriate interpretation of chest X-rays should be a cause for concern.

In order to enhance inter-rater reliability, the ARDS Definition Task Force (“the panel”) has developed a set of chest radiographs judged to be consistent, inconsistent, or equivocal for the diagnosis of ARDS [3]. The question of whether these chest radiographs could improve the accuracy of ARDS diagnoses has not been resolved.

Therefore, we performed a prospective study to examine the effect of the training material developed by the panel on the accuracy and consistency of chest radiograph interpretation by intensivists for diagnosing ARDS in accordance with the Berlin definition.

## Methods

### Source of chest radiographs

We used the set of 12 chest radiographs developed by the panel [3] for the diagnosis of ARDS; these radiographs were classified into the consistent (*n* = 4), inconsistent (*n* = 4), and equivocal (*n* = 4) categories and were provided without any additional clinical information.

### Study protocol

This study was conducted during a 3-month period in 24 intensive care units (ICUs) of the China Critical Care Clinical Trials Group (CCCCTG). All intensivists working in the participating ICUs were eligible for the study. The exclusion criteria were awareness of the study plan (i.e., involvement in study design and/or conception), a planned rotation outside the ICU within 3 months (i.e., the end of the study), and previous review of the set of 12 chest radiographs.

A survey questionnaire accompanied by instructions was sent via e-mail to contact persons in participating ICUs and then distributed to all participating intensivists, who were asked to independently complete the questionnaire within 1 hour after providing informed consent. Participants were informed of the aim of the study, but were not aware of the precise methodology including the subsequent training and a second survey 2 months later. The questionnaire contained all 12 chest radiographs provided by the panel. In addition, the Berlin definition of ARDS, including the radiographic diagnostic criterion, was also attached at the end of the questionnaire for clarification. Participants reported responses of “consistent”, which indicated that a chest radiograph satisfied the Berlin definition of ARDS; “inconsistent”, which indicated radiologic abnormalities suggestive of a disorder other than ARDS; and “equivocal”, which indicated uncertainty regarding the exact cause of the observed radiologic abnormalities. Data on the characteristics of the respondent, including age, sex, status of education, type of ICU, appointment, working experience, and any other professional background, were also recorded. All questionnaires were completed and returned to the principal investigator via e-mail within 1 week.

After receiving completed questionnaires, the principal investigator sent an e-mail to the contact person at each ICU. The e-mail comprised the reference paper with supplementary material including 12 radiographs from the panel [3], as well as a training slide with the principle and rationale of radiographic interpretation based on the above materials. All materials were translated into Chinese by the principal investigator for better comprehension. The accuracy of the translation was validated by back translation. The contact person at each ICU was also required to attend a training course within 1 week after receiving the e-mail. During the training course, which lasted for at least 1.5 hours, the contact person explained the diagnostic rationale using the training slides, followed by an in-depth discussion to come to a consensus for all 12 radiographs.

After 2 months, all participants were asked to complete the second questionnaire, which included the same 12 chest radiographs, although in a different sequence. The questionnaire also recorded the age, sex, and appointment of the respondent in order to match the interpretation of the chest radiographs to the same respondent.

The study protocol was approved by the institutional review board of Fuxing Hospital, Capital Medical University, and has been registered at ClinicalTrials.Gov (NCT0170466).

### Supplemental survey to test the memory of the respondents

After completing the experiment described above, we performed another survey to differentiate between memory effects and true interpretations of individual chest radiographs. A convenience sample of 24 intensivists who had not participated in the aforementioned experiment was selected from all participating ICUs. An e-mail containing the same 12 chest radiographs was sent to these participants, who remained unaware of the objective of the survey. Two months later, another e-mail containing the same 12 chest radiographs and 12 different chest radiographs was sent to these 24 intensivists. The participants were asked the following question: “Have you ever reviewed this chest radiograph before?” Each chest radiograph received an answer of “Yes” or “No” from each individual intensivist.

### Statistical analysis

The radiographic diagnosis by the panel was used as the “gold standard” [3]. The accuracy of the reports by the participating intensivists with respect to the “gold standard” was assessed for overall accuracy (i.e., percentage of chest radiographs with the correct diagnosis), sensitivity and specificity for each diagnostic category (i.e., consistent, equivocal, and inconsistent) [7]. In particular, when calculating sensitivity and specificity for each diagnostic category (e.g., consistent), data for the other two categories (i.e., equivocal and inconsistent) were combined and treated as one diagnostic group. Youden’s J statistic, calculated as sensitivity + specificity – 1, was used to compare the overall performance of the diagnosis [8]. The inter-rater agreement among all participating intensivists was assessed by overall agreement, chance-corrected agreement (Fleiss’s kappa) [9], and intraclass correlation coefficient (ICC) with a two-way random model [10].

Student’s *t* tests and Mann–Whitney *U* tests were employed when comparing two groups, and univariate analysis of variance using the F statistic was employed to test group (>2) comparisons. The Bonferroni post hoc test was used for multiple comparisons. The Z test developed by Fisher was used to compare the ICC between groups [11]. Categorical variables were reported as a percentage of the group from which they were derived and were compared using the chi-square test or Fisher’s exact test when appropriate.

We also compared the diagnostic accuracy and inter-rater agreement on the ARDS radiographs among different subgroups categorized by age, sex, appointment, professional degree, years of medical practice, type of ICU, years of ICU practice, and other professional background.

For the supplemental study examining the memory of the respondents, we reported overall accuracy, and we compared the accuracy of the chest radiographs that the intensivists did and did not review previously.

## Results

### Characteristics of the participating intensivists

There were 400 intensivists in the 24 participating ICUs, and 110 were excluded from the study. The reasons for exclusion included planned rotation outside the ICU (*n* = 66), awareness of the study design (*n* = 24), previous review of the set of 12 chest radiographs (*n* = 18), refused participation (*n* = 1), and unknown (*n* = 1). Moreover, four intensivists from two hospitals did not respond to the second survey, thus leaving 286 participants in the final analysis.

Among the 286 respondents, the median age was 32.5 years, and 163 (57.0%) were male (Table 1). There were 118 (41.3%) residents, 101 (35.3%) junior attending physicians, and 67 (23.4%) senior attending physicians. More than 60% of the respondents were working in general ICUs, and approximately 40% did not have a background in fields outside of critical care. The respondents had a median length of experience in critical care practice of 5 years (range, 0 to 23 years).

**Table 1:** Characteristics of 286 participating intensivists

Characteristic	All (*n* = 286)
Age, median (IQR)	32.5 (30, 39)
Male sex, n (%)	163 (57.0)
Professional degree, n (%)	
Doctorate	60 (21.0)
Master	161 (56.3)
Bachelor	64 (22.4)
Other	1 (0.3)
Position, n (%)	
Resident	118 (41.3)
Junior attending	101 (35.3)
Senior attending	67 (23.4)
Years of medical practice, median (IQR)	8 (4, 14.25)
Years of critical care practice, median (IQR)	5 (2, 10)
Type of intensive care unit, n (%)	
General	182 (63.6)
Surgical	38 (13.3)
Emergency	66 (23.1)
Other background than critical care, n (%)	
None	118 (41.3)
Medicine	82 (28.7)
Surgery	42 (14.7)
Emergency	16 (5.6)
Anesthesia	21 (7.3)
Other	7 (2.4)
Years of practicing other background, median (IQR)	2 (0, 5)

*IQR* interquartile range

### Accuracy of the radiographic diagnosis of ARDS

**Fig. 1:**
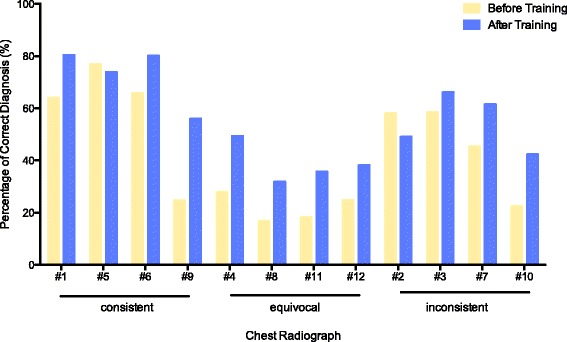
Diagnostic accuracies for 12 chest radiographs for the 286 participating intensivists before and after training. Consistent, chest radiographs consistent with ARDS, as judged by the panel; equivocal, chest radiographs equivocal for ARDS, as judged by the panel; inconsistent, chest radiographs inconsistent with ARDS, as judged by the panel

**Fig. 2:**
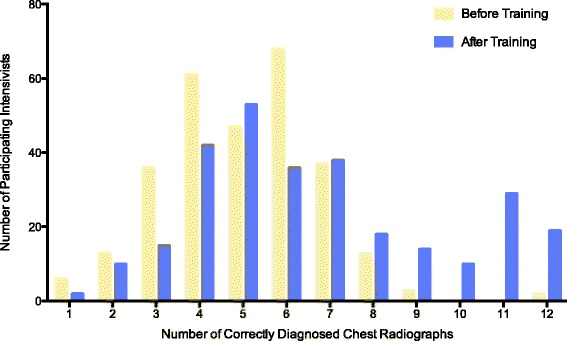
Distribution of 286 intensivists by numbers of correctly diagnosed chest radiographs before and after training

Before training, the 286 participating intensivists made a correct diagnosis in 5.0 ± 1.8 chest radiographs, including 2.3 ± 1.1 consistent, 0.9 ± 1.0 equivocal, and 1.9 ± 1.1 inconsistent results. After training, the number of correctly diagnosed chest radiographs remained low, despite an increase to 6.6 ± 2.8 radiographs (a mean difference of 1.6, with a 95% confidence interval [CI] of 1.2 to 2.0, *p* < 0.001), including 2.9 ± 1.1 consistent radiographs, 1.6 ± 1.4 equivocal radiographs, and 2.2 ± 1.2 inconsistent radiographs (Table 2, Figs. 1 and 2). This result corresponded to an improvement in overall accuracy from 42.0 ± 14.8% to 55.3 ± 23.4% (a mean difference of 13.3%, with a 95% CI of 10.2 to 16.5%, *p* < 0.001). In particular, we observed increased, unchanged, and decreased overall diagnostic accuracy in 156 (54.5%), 50 (17.5%), and 80 (28.0%) participating intensivists, respectively.

**Table 2:** Accuracy of radiographic diagnosis of acute respiratory distress syndrome among 286 participating intensivists

Variables	Before training	After training	Mean difference (95%CI)	*p* value
All chest radiographs				
Number of correctly diagnosed cases	5.0 ± 1.8	6.6 ± 2.8	1.6 (1.2 to 2.0)	<0.001
Overall accuracy^a^	42.0 ± 14.8%	55.3 ± 23.4%	13.3% (10.2 to 16.5%)	<0.001
Chest radiographs consistent with ARDS				
Number of correctly diagnosed cases	2.3 ± 1.1	2.9 ± 1.1	0.6 (0.4 to 0.8)	<0.001
Diagnostic accuracy	57.5 ± 27.5%	72.5 ± 27.5%	15.0% (10.0 to 20.0%)	<0.001
Sensitivity	0.579 ± 0.285	0.726 ± 0.279	0.147 (0.105 to 0.188)	<0.001
Specificity	0.734 ± 0.209	0.783 ± 0.190	0.049 (0.018 to 0.080)	0.002
Youden’s J statistic	0.313 ± 0.347	0.509 ± 0.370	0.196 (0.140 to 0.253)	<0.001
Chest radiographs equivocal for ARDS				
Number of correctly diagnosed cases	0.9 ± 1.0	1.6 ± 1.4	0.7 (0.5 to 0.8)^*^	<0.001
Diagnostic accuracy	22.5 ± 25.0%	40.0% ± 35.0%	17.5% (12.5 to 20.0%)	<0.001
Sensitivity	0.219 ± 0.245	0.387 ± 0.339	0.168 (0.125 to 0.211)	<0.001
Specificity	0.824 ± 0.153	0.823 ± 0.168	−0.002 (−0.025 to 0.022)	0.883
Youden’s J statistic	0.044 ± 0.258	0.210 ± 0.407	0.166 (0.116 to 0.216)	<0.001
Chest radiographs inconsistent with ARDS				
Number of correctly diagnosed cases	1.9 ± 1.1	2.2 ± 1.2	0.3 (0.2 to 0.5)	<0.001
Diagnostic accuracy	47.5 ± 27.5%	55.0 ± 30.0%	7.5% (5.0 to 12.5%)	<0.001
Sensitivity	0.462 ± 0.285	0.547 ± 0.302	0.086 (0.040 to 0.131)	<0.001
Specificity	0.572 ± 0.213	0.724 ± 0.204	0.153 (0.122 to 0.183)	<0.001
Youden’s J statistic	0.033 ± 0.301	0.271 ± 0.418	0.238 (0.181 to 0.295)	<0.001

*ARDS* acute respiratory distress syndrome, *CI* confidence interval

^*^ *p* = 0.026 vs. chest radiographs inconsistent with ARDS

^a^Accuracy defined as percentage of chest radiographs interpreted correctly

Among the three categories, the diagnostic accuracy was highest for the consistent category, moderate for the inconsistent category, and lowest for the equivocal category, as demonstrated by the number of correctly diagnosed cases, diagnostic accuracy, and Youden’s J statistic (*p* < 0.001) (Table 2). This result was true both before and after training, except that Youden’s J statistic was similar for the equivocal and inconsistent categories before training (*p* = 0.593). Moreover, the improvement of diagnostic accuracy was more remarkable in the equivocal category (*p* = 0.024; post hoc test demonstrated significant difference among the equivocal category vs. inconsistent category) (Table 2).

For the consistent and inconsistent categories, both diagnostic sensitivity and specificity increased significantly after training. In comparison, for the equivocal category, specificity remained unchanged (0.824 ± 0.153 vs. 0.823 ± 0.168, *p* = 0.883) despite a significant improvement in sensitivity (0.219 ± 0.245 vs. 0.387 ± 0.339, *p* < 0.001) after training (Table 2). Subgroup analyses suggested that senior physicians (i.e., those with more years of medical or intensive care practice) exhibited a marginally, despite statistically significant, better diagnostic accuracy. In addition, the aforementioned improvement in diagnostic accuracy was consistent across all subgroups, including subgroups divided by age, sex, appointment, professional degree, years of medical practice, type of ICU, years of ICU practice, and other professional background (Additional file 1: Table S1).

### Inter-rater agreement on the radiographic diagnosis of ARDS

Inter-rater agreement was poor among the 286 participating intensivists. Comparisons of pre-training and post-training results revealed that training did not have any impact on inter-rater agreement, as suggested by insignificant changes in overall agreement (0.450 [95% CI, 0.397 to 0.504] vs. 0.461 [95% CI, 0.387 to 0.504], *p* = 0.792), Fleiss’s kappa (0.133 [95% CI, 0.058 to 0.207] vs. 0.178 [95% CI, 0.086 to 0.270], *p* = 0.405), and ICC (0.219 [95% CI, 0.122 to 0.449] vs. 0.276 [95% CI, 0.159 to 0.525], *p* = 0.470). There was no statistically significant difference in inter-rater agreement between any subgroups. In addition, we observed no improvement in inter-rater agreement after training in any subgroups (Additional file 1: Table S2).

### Supplemental survey to test the memory of the respondents

The overall accuracy, i.e., the accuracy of respondents correctly identifying all 24 chest radiographs, was 51.9 ± 9.8%, with no significant difference between the set of 12 chest radiographs previously reviewed and those not previously reviewed (55.2 ± 14.9% vs. 48.6 ± 14.9%, *p* = 0.165).

## Discussion

To our knowledge, this is the first study to explore the reliability of the newly proposed Berlin radiographic definition of ARDS. We found that the accuracy of radiographic diagnosis of ARDS remained poor even after training with the set of chest radiographs developed by the panel, although significant improvement was observed based on overall accuracy and Youden’s J statistic; training did not change inter-rater agreement.

Only two previous studies reported the inter-rater variability in applying the AECC radiographic criterion for ARDS [4, 5]. Rubenfeld et al. reported moderate inter-rater agreement (kappa 0.55) among 21 experts who reviewed 28 randomly selected chest radiographs [4]. Meade et al. also found that intensivists without formal consensus training could achieve moderate levels of agreement (kappa 0.72 to 0.88) [5]. While recognizing the aforementioned limitations, the panel retained bilateral opacities consistent with pulmonary edema on chest radiographs as the defining criterion for ARDS, but they explicitly specified that the above abnormalities could not be fully explained by effusions, lobar/lung collapse, or nodules/masses [2]. The panel expected to enhance inter-rater reliability through the inclusion of a set of chest radiographs and called for evaluation of the reliability of case identification based on the Berlin radiographic criterion [3].

Our study differed from previous studies. First, we used the set of 12 chest radiographs judged by the panel to be the “gold standard”; this approach allowed us to evaluate diagnostic accuracy, an assessment that was impossible in prior studies due to the lack of a “gold standard” [4, 5]. The panel was composed of international experts in ARDS who were actively involved in the development of the Berlin definition of ARDS [2]. The consensus reached by the panel regarding the radiographic diagnosis of the 12 reference chest radiographs might therefore represent the best available judgement. Moreover, it was the expectation of the panel that the radiographic diagnosis of ARDS might be standardized through the use of this training material. Second, because the diagnosis of ARDS and the decision to enroll patients in clinical trials are frequently made by clinicians at the bedside, we believe that our study sample (of 286 participating intensivists from multiple institutions) might be more representative of inter-rater variability in routine clinical practice than samples used in prior studies in which inter-rater agreement was assessed among either international experts or a small number of intensivists [4, 5]. We also included intensivists at various stages of training, allowing us to explore the influence of such training on the degree of improvement.

However, the main results of our study were disappointing, with the accuracy of radiographic diagnosis of ARDS barely greater than 50%, even after training. This finding suggests that, even with the new definition of ARDS and training materials, the interpretation of chest radiographs for ARDS remains problematic. The ability to correctly interpret chest radiographs was recognized as one of the core competencies for an international training program in intensive care medicine in the European Union [12] and mainland China [13]; this competency could only be acquired after reading hundreds of normal and abnormal chest radiographs [14] or taking a training course [15]. Therefore, it appears unrealistic to expect significant improvement in intensivists’ global skills with respect to the interpretation of chest radiographs after reviewing only 12 chest radiographs. The set of 12 chest radiographs developed by the panel should only be regarded as examples that can be used as a basis for developing final training materials that include a larger set of chest radiographs with diagnoses confirmed by experienced radiologists. In addition, methods other than visual inspection might merit further investigation. For example, Herasevich et al. reported that electronic ARDS screening based on real-time query of chest X-ray readings with arterial blood gas values demonstrated excellent sensitivity of 96% and moderate specificity of 89% [16].

We found that intensivists performed significantly better at identifying chest radiographs consistent with ARDS than those inconsistent with or equivocal for ARDS. Prior studies have demonstrated that atelectasis, pleural effusion, vascular redistribution, and overlying monitoring equipment that obscures the pulmonary parenchyma are perceived by experts as problematic [4] and can lead to difficult and often misleading interpretations of chest radiographs. Therefore, if more extensive training materials that focus more on the aforementioned difficulties were available, it might be possible to further improve both diagnostic accuracy and inter-rater agreement.

Clinical consequence of our findings in the management of ARDS remains uncertain due to the lack of specific therapies apart from lung-protective ventilator strategy. However, improved diagnostic accuracy and inter-rater agreement with regards to ARDS radiographic interpretation are crucial to the enrollment of more homogeneous patient population in clinical studies. Therefore, a multifaceted strategy may be important when designing relevant training courses. Such strategy should include, but not be limited to, more iterative series of training sessions, more sample radiographs and accompanied instruction for radiographic interpretation (especially inconsistent or equivocal categories), involvement of radiologists as instructors, adoption of an interactive learning approach, and even neural networks and deep learning. Moreover, our findings strongly suggest that these training courses should target both junior and senior intensivists.

Our study has several limitations. First, the number of radiographs reviewed was quite small compared with the 778 radiographs assessed by Meade [5]. Nevertheless, these were the only available radiographs with the consensus judgement by the panel that could be considered as a “gold standard”. In addition, this might be partially overcome by the large number of participants in our study. Second, the context in which the present study was performed was not a real-life situation. For example, series of chest radiographs were not available; such series can be important for delineating the obscuring effects of pleural effusion or overlying monitoring equipment. However, many clinical trials of ARDS often exclude patients with ARDS for more than 36 or 48 hours [17–20]; typically, only one or two chest radiographs are available within this short time window. Finally, memory effects could not be completely excluded because the participants reviewed the same set of chest radiographs in the verification survey. The fact that the overall diagnostic accuracy improved, while the inter-rater agreement did not, might also suggest the possibility of a memory effect. However, the results of the supplemental survey, despite using a different study population, did not support the above hypothesis. It is also noteworthy that, even if taking into account the above confounding factors, the diagnostic accuracy as well as inter-rater agreement still remained poor after training.

## Conclusions

Our results demonstrated that both the accuracy and inter-rater agreement of the radiographic diagnosis of ARDS were poor, even after training with the set of 12 chest radiographs developed by the panel. As a result, this set of chest radiographs should be regarded only as an example that may be used for the development of future training materials. Further investigations are needed to explore a more effective approach to improve the accuracy and inter-rater reliability of ARDS radiographic interpretation.

## Additional file

Additional file 1: Table S1. Accuracy of radiographic diagnosis of acute respiratory distress syndrome across subgroups. **Table S2.** Inter-rater agreement in applying radiographic definition of acute respiratory distress syndrome across subgroups. (DOCX 102 kb)

## Abbreviations

AECC: American-European Consensus Conference
ARDS: Acute respiratory distress syndrome
CCCCTG: China Critical Care Clinical Trial Group
CI: Confidence interval
ICC: Intraclass correlation coefficient
ICUs: Intensive care units
