# Supplementary material for: Does training improve diagnostic accuracy and inter-rater agreement in applying the Berlin radiographic definition of acute respiratory distress syndrome? A multicenter prospective study
Source: Crit Care. 2017 Jan 20;21:12. doi: 10.1186/s13054-017-1606-4 (PMC5251343; doi:10.1186/s13054-017-1606-4)
Supplement: Additional file 1: Table S1. — Accuracy of radiographic diagnosis of acute respiratory distress syndrome across subgroups. Table S2. Inter-rater agreement in applying radiographic definition of acute respiratory distress syndrome across subgroups. (DOCX 102 kb) [file 13054_2017_1606_MOESM1_ESM.docx]

**Does training improve diagnostic accuracy and inter-rater agreement in applying the Berlin radiographic definition of acute respiratory distress syndrome? A multicentre prospective study**

Jin-Min Peng, MD^1^; Chuan-Yun Qian, MD^2^; Xiang-You Yu, MD^3^; Ming-Yan Zhao, MD^4^; Shu-Sheng Li, MD^5^; Xiao-Chun Ma, MD^6^; Yan Kang, MD^7^; Fa-chun Zhou, MD^8^; Zhen-Yang He, MD^9^; Tie-He Qin, MD^10^; Yong-Jie Yin, MD^11^; Li Jiang, MD^12^; Zhen-Jie; Hu, MD^13^; Ren-Hua Sun, MD^14^; Jian-Dong Lin, MD^15^; Tong Li, MD^16^; Da-Wei Wu, MD^17^; You-Zhong An, MD^18^; Yu-Hang Ai, MD^19^; Li-Hua Zhou, MD^20^; Xiang-Yuan Cao, MD^21^; Xi-Jing Zhang, MD^22^; Rong-Qing Sun, MD^23^; Er-Zhen Chen, MD^24^; Bin Du, MD^1^ ； on behalf of the China Critical Care Clinical Trial Group (CCCCTG)

**Table S1** Accuracy of radiographic diagnosis of acute respiratory distress syndrome across subgroups

|  | Overall Accuracy | | Mean difference |  |
| --- | --- | --- | --- | --- |
| Variables | Before Training | After Training | (95% Confidence Interval) | *P* value |
| Age |  |  |  |  |
| < 32.5 years (n = 143) | 40.7 ± 14.6% | 53.4 ± 22.7% | 12.7% (8.4 to 17.0%) | < 0.001 |
| ≥ 32.5 years (n = 143) | 43.3 ± 14.9% | 57.3 ± 24.1% | 14.0% (9.4 to 18.6%) | < 0.001 |
| Sex |  |  |  |  |
| Male (n = 163) | 43.6 ± 15.1% | 56.6 ± 23.0% | 13.0% (8.8 to 17.3%) | < 0.001 |
| Female (n = 123) | 39.9 ± 14.1%* | 53.7 ± 24.0% | 13.8% (9.1 to 18.4%) | < 0.001 |
| Professional degree |  |  |  |  |
| Doctorate (n = 60) | 43.3 ± 12.7% | 51.3 ± 22.7% | 7.9% (1.2 to 14.6%) | 0.021 |
| Master (n = 161) | 40.2 ± 14.8% | 56.1 ± 23.9% | 15.8% (11.6 to 20.0%) | < 0.001 |
| Bachelor (n = 64) | 45.3 ± 16.1% | 57.7 ± 22.9% | 12.4% (5.8 to 18.9%) | < 0.001 |
| Appointment |  |  |  |  |
| Resident (n = 118) | 40.6 ± 14.6% | 53.8 ± 24.0% | 13.2% (8.2 to 18.2%) | < 0.001 |
| Junior attending (n = 101) | 42.2 ± 14.0% | 56.8 ± 24.1% | 14.6% (9.1 to 20.1%) | < 0.001 |
| Senior attending (n = 67) | 44.2 ± 16.0% | 55.8 ± 21.4% | 11.7% (5.8 to 17.6%) | < 0.001 |
| Years of medical practice |  |  |  |  |
| < 8 years (n = 136) | 40.0 ± 15.1% | 54.2 ± 23.6% | 14.2% (9.6 to 18.7%) | < 0.001 |
| ≥ 8 years (n = 150) | 43.8 ± 14.3%† | 56.4 ± 23.3% | 12.6% (8.3% to 16.9%) | < 0.001 |
| Years of critical care practice |  |  |  |  |
| < 5 years (n = 131) | 40.1 ± 15.3% | 53.9 ± 24.2% | 13.9% (8.9 to 18.8%) | < 0.001 |
| ≥ 5 years (n = 155) | 43.6% ± 14.2%‡ | 56.5 ± 22.8% | 12.9% (8.9 to 16.9%) | < 0.001 |
| Type of intensive care unit |  |  |  |  |
| General (n = 182) | 43.2 ± 14.4% | 55.1 ± 22.2% | 11.9% (8.2 to 15.6%) | < 0.001 |
| Surgical (n = 38) | 37.7 ± 13.2% | 43.2 ± 15.6% | 5.5% (0.1 to 10.9%) | 0.047 |
| Emergency (n = 66) | 41.0 ± 16.4% | 62.9 ± 27.5% | 21.8% (13.7 to 30.0%) | < 0.001 |
| Other background |  |  |  |  |
| None (n = 118) | 41.5 ± 15.6% | 56.4 ± 24.1% | 14.8% (10.0 to 19.7%) | < 0.001 |
| Medicine (n = 82) | 41.5 ± 14.2% | 53.5 ± 22.9% | 12.0% (6.1 to 17.9%) | < 0.001 |
| Surgery (n = 42) | 41.3 ± 13.9% | 55.8 ± 23.9% | 14.5% (6.4 to 22.6%) | 0.001 |
| Emergency (n = 16) | 46.9 ± 18.7% | 67.2 ± 26.6% | 20.3% (1.8 to 38.8%) | 0.033 |
| Anesthesia (n = 21) | 45.2 ± 11.7% | 46.0 ± 12.5% | 0.8% (-6.2 to 7.8%) | 0.815 |
| Other (n = 7) | 39.3 ± 11.5% | 58.3 ± 26.8% | 19.0% (-10.4 to 48.5%) | 0.164 |
| Year of practicing other background |  |  |  |  |
| < 2 years (n = 140) | 41.0 ± 14.6% | 55.6 ± 23.8% | 14.6% (10.1 to 19.2%) | < 0.001 |
| ≥ 2 years (n = 146) | 43.0 ± 14.9% | 55.1 ± 23.1% | 12.1% (7.8 to 16.4%) | < 0.001 |

**p* = 0.038 vs. male sex; †p = 0.031 vs. years of medical practice < 8 years; ‡p = 0.044 vs. years of critical care practice < 5 years.

**Table S2** Inter-rater agreement in applying radiographic definition of acute respiratory distress syndrome across subgroups

|  | Overall Agreement | | | Fleiss’s Kappa | | | Intra-class Correlation Coefficient | | |
| --- | --- | --- | --- | --- | --- | --- | --- | --- | --- |
| Variable | Before | After | *P* value | Before | After | *P* value | Before | After | *P* value |
| Overall (n = 286) | 0.450 (0.397 to 0.504) | 0.461 (0.387 to 0.536) | 0.792 | 0.133 (0.058 to 0.207) | 0.178 (0.086 to 0.270) | 0.405 | 0.219 (0.122 to 0.449) | 0.276 (0.159 to 0.525) | 0.470 |
| Age |  |  |  |  |  |  |  |  |  |
| < 32.5 years (n = 143) | 0.459 (0.401 to 0.517) | 0.452 (0.381 to 0.522) | 0.865 | 0.132 (0.053 to 0.212) | 0.162 (0.072 to 0.251) | 0.583 | 0.209 (0.114 to 0.437) | 0.255 (0.143 to 0.500) | 0.684 |
| ≥ 32.5 years (n = 143) | 0.442 (0.389 to 0.495) | 0.473 (0.390 to 0.556) | 0.491 | 0.131 (0.058 to 0.204) | 0.197 (0.096 to 0.299) | 0.245 | 0.227 (0.125 to 0.463) | 0.301 (0.175 to 0.557) | 0.505 |
| Sex |  |  |  |  |  |  |  |  |  |
| Male (n = 163) | 0.454 (0.397 to 0.512) | 0.478 (0.396 to 0.560) | 0.596 | 0.133 (0.052 to 0.214) | 0.195 (0.095 to 0.295) | 0.294 | 0.223 (0.122 to 0.456) | 0.290 (0.168 to 0.544) | 0.521 |
| Female (n = 123) | 0.447 (0.396 to 0.498) | 0.441 (0.375 to 0.507) | 0.874 | 0.134 (0.064 to 0.203) | 0.155 (0.071 to 0.239) | 0.673 | 0.215 (0.117 to 0.447) | 0.257 (0.144 to 0.503) | 0.730 |
| Professional Degree |  |  |  |  |  |  |  |  |  |
| Doctorate (n = 60) | 0.492 (0.426 to 0.557) | 0.446 (0.371 to 0.522) | 0.312 | 0.191 (0.090 to 0.292) | 0.154 (0.070 to 0.238) | 0.536 | 0.316 (0.181 to 0.578) | 0.241 (0.130 to 0.488) | 0.664 |
| Master (n = 161) | 0.435 (0.383 to 0.488) | 0.460 (0.396 to 0.524) | 0.507 | 0.111 (0.039 to 0.183) | 0.178 (0.094 to 0.262) | 0.184 | 0.188 (0.101 to 0.404) | 0.279 (0.160 to 0.530) | 0.392 |
| Bachelor (n = 64) | 0.460 (0.390 to 0.530) | 0.486 (0.387 to 0.584) | 0.579 | 0.138 (0.057 to 0.219) | 0.209 (0.089 to 0.329) | 0.286 | 0.227 (0.121 to 0.468) | 0.315 (0.181 to 0.577) | 0.600 |
| Appointment |  |  |  |  |  |  |  |  |  |
| Resident (n = 118) | 0.459 (0.402 to 0.516) | 0.447 (0.384 to 0.509) | 0.574 | 0.127 (0.044 to 0.210) | 0.158 (0.075 to 0.241) | 0.565 | 0.202 (0.109 to 0.427) | 0.243 (0.135 to 0.486) | 0.744 |
| Junior attending (n = 101) | 0.452 (0.397 to 0.507) | 0.479 (0.394 to 0.564) | 0.561 | 0.123 (0.049 to 0.197) | 0.199 (0.089 to 0.308) | 0.210 | 0.208 (0.112 to 0.438) | 0.302 (0.174 to 0.560) | 0.481 |
| Senior attending (n = 67) | 0.443 (0.395 to 0.492) | 0.463 (0.373 to 0.553) | 0.668 | 0.155 (0.083 to 0.228) | 0.182 (0.081 to 0.283) | 0.634 | 0.262 (0.145 to 0.513) | 0.292 (0.165 to 0.550) | 0.854 |
| Years of medical practice |  |  |  |  |  |  |  |  |  |
| < 8 years (n = 136) | 0.461 (0.403 to 0.518) | 0.455 (0.385 to 0.526) | 0.884 | 0.126 (0.046 to 0.206) | 0.169 (0.078 to 0.260) | 0.438 | 0.198 (0.107 to 0.420) | 0.264 (0.149 to 0.512) | 0.570 |
| ≥ 8 years (n = 150) | 0.445 (0.385 to 0.506) | 0.468 (0.382 to 0.554) | 0.628 | 0.140 (0.063 to 0.217) | 0.188 (0.083 to 0.293) | 0.414 | 0.241 (0.135 to 0.482) | 0.288 (0.166 to 0.542) | 0.665 |
| Years of critical care practice |  |  |  |  |  |  |  |  |  |
| < 5 years (n = 131) | 0.451 (0.395 to 0.507) | 0.450 (0.382 to 0.518) | 0.980 | 0.118 (0.039 to 0.196) | 0.158 (0.075 to 0.241) | 0.446 | 0.194 (0.104 to 0.415) | 0.241 (0.134 to 0.482) | 0.693 |
| ≥ 5 years (n = 155) | 0.451 (0.393 to 0.509) | 0.471 (0.391 to 0.552) | 0.659 | 0.145 (0.064 to 0.226) | 0.195 (0.093 to 0.298) | 0.398 | 0.243 (0.136 to 0.484) | 0.307 (0.179 to 0.564) | 0.546 |
| Type of intensive care unit |  |  |  |  |  |  |  |  |  |
| General (n = 182) | 0.464 (0.409 to 0.519) | 0.465 (0.384 to 0.546) | 0.982 | 0.158 (0.076 to 0.240) | 0.186 (0.083 to 0.289) | 0.640 | 0.252 (0.149 to 0.509) | 0.297 (0.173 to 0.552) | 0.645 |
| Surgical (n = 38) | 0.484 (0.395 to 0.573) | 0.440 (0.393 to 0.487) | 0.339 | 0.140 (0.033 to 0.248) | 0.112 (0.054 to 0.170) | 0.615 | 0.219 (0.111 to 0.463) | 0.168 (0.080 to 0.389) | 0.825 |
| Emergency (n = 66) | 0.413 (0.355 to 0.470) | 0.496 (0.425 to 0.566) | 0.046 | 0.079 (0.012 to 0.146) | 0.235 (0.150 to 0.321) | 0.002 | 0.128 (0.062 to 0.313) | 0.295 (0.167 to 0.553) | 0.325 |
| Other background |  |  |  |  |  |  |  |  |  |
| None (n = 118) | 0.453 (0.394 to 0.512) | 0.460 (0.389 to 0.531) | 0.867 | 0.135 (0.051 to 0.219) | 0.178 (0.089 to 0.267) | 0.442 | 0.226 (0.124 to 0.462) | 0.278 (0.159 to 0.531) | 0.674 |
| Medicine (n = 82) | 0.453 (0.404 to 0.501) | 0.454 (0.376 to 0.532) | 0.981 | 0.146 (0.076 to 0.215) | 0.172 (0.071 to 0.273) | 0.644 | 0.245 (0.134 to 0.490) | 0.286 (0.162 to 0.542) | 0.782 |
| Surgery (n = 42) | 0.432 (0.378 to 0.486) | 0.464 (0.380 to 0.548) | 0.483 | 0.076 (0.009 to 0.144) | 0.162 (0.069 to 0.256) | 0.109 | 0.116 (0.051 to 0.299) | 0.232 (0.121 to 0.480) | 0.597 |
| Emergency (n = 16) | 0.449 (0.358 to 0.539) | 0.531 (0.413 to 0.649) | 0.236 | 0.123 (0.017 to 0.229) | 0.290 (0.126 to 0.454) | 0.071 | 0.291 (0.142 to 0.569) | 0.395 (0.220 to 0.670) | 0.763 |
| Anesthesia (n = 21) | 0.471 (0.396 to 0.547) | 0.453 (0.366 to 0.541) | 0.733 | 0.160 (0.056 to 0.265) | 0.158 (0.042 to 0.274) | 0.978 | 0.255 (0.124 to 0.521) | 0.240 (0.115 to 0.504) | 0.962 |
| Other (n = 7) | 0.476 (0.314 to 0.638) | 0.425 (0.281 to 0.569) | 0.614 | 0.158 (0.000 to 0.370) | 0.128 (0.000 to 0.314) | 0.819 | 0.314 (0.115 to 0.623) | 0.186 (0.026 to 0.492) | 0.847 |
| Year of practicing other background |  |  |  |  |  |  |  |  |  |
| < 2 years (n = 140) | 0.456 (0.398 to 0.514) | 0.456 (0.389 to 0.523) | > 0.999 | 0.135 (0.055 to 0.214) | 0.173 (0.090 to 0.257) | 0.469 | 0.217 (0.119 to 0.449) | 0.270 (0.154 to 0.520) | 0.641 |
| ≥ 2 years (n = 146) | 0.444 (0.392 to 0.496) | 0.465 (0.382 to 0.549) | 0.637 | 0.129 (0.056 to 0.201) | 0.181 (0.076 to 0.285) | 0.366 | 0.218 (0.120 to 0.450) | 0.281 (0.161 to 0.533) | 0.570 |
